# Disruption of the *Schizosaccharomyces japonicus lig4* Disturbs Several Cellular Processes and Leads to a Pleiotropic Phenotype

**DOI:** 10.3390/jof9050550

**Published:** 2023-05-10

**Authors:** Lajos Acs-Szabo, Laszlo Attila Papp, Szonja Takacs, Ida Miklos

**Affiliations:** Department of Genetics and Applied Microbiology, Faculty of Science and Technology, University of Debrecen, Egyetem tér 1, 4032 Debrecen, Hungary

**Keywords:** *Schizosaccharomyces japonicus*, *lig4* gene, NHEJ, gene targeting, RNA sequencing, TOR pathway

## Abstract

Gene targeting is a commonly used method to reveal the function of genes. Although it is an attractive tool for molecular studies, it can frequently be a challenge because its efficiency can be low and it requires the screening of a large number of transformants. Generally, these problems originate from the elevated level of ectopic integration caused by non-homologous DNA end joining (NHEJ). To eliminate this problem, NHEJ-related genes are frequently deleted or disrupted. Although these manipulations can improve gene targeting, the phenotype of the mutant strains raised the question of whether mutations have side effects. The aim of this study was to disrupt the *lig4* gene in the dimorphic fission yeast, *S. japonicus*, and investigate the phenotypic changes of the mutant strain. The mutant cells have shown various phenotypic changes, such as increased sporulation on complete medium, decreased hyphal growth, faster chronological aging, and higher sensitivity to heat shock, UV light, and caffeine. In addition, higher flocculation capacity has been observed, especially at lower sugar concentrations. These changes were supported by transcriptional profiling. Many genes belonging to metabolic and transport processes, cell division, or signaling had altered mRNA levels compared to the control strain. Although the disruption improved the gene targeting, we assume that the *lig4* inactivation can cause unexpected physiological side effects, and we have to be very careful with the manipulations of the NHEJ-related genes. To reveal the exact mechanisms behind these changes, further investigations are required.

## 1. Introduction

DNA ligases catalyze the joining of two DNA molecules. One of them is LIG4, which can be found in various species, from yeasts to humans [1,2,3,4,5]. Several studies have proved that LIG4 proteins are essential components of the non-homologous end joining (NHEJ) mechanism, which repairs the double-strand breaks of DNA [1,5,6,7]. Double-strand breaks (DSBs) are severe forms of mutations that can be repaired by different mechanisms. In the case of the NHEJ mechanism, the KU70 and 80 proteins bind and process the DNA ends, which is followed by ligation carried out by the LIG4-XRCC4 complex [8,9]. The mutations of these genes lead to decreased NHEJ and increased homologous recombination (HR) [10]. 

In addition, mutations in the human LIG4 gene are associated with various diseases. Patients with ligase IV syndrome suffer from a broad spectrum of clinical features, from developmental delay and growth retardation to skin anomalies or malignancy predisposition [11,12]. Abnormal LIG4 expression has been detected in prostate or colorectal cancer [13,14], and LIG4 mutations have also been found in Omenn syndrome, which is a form of severe combined immunodeficiency [15]. Other studies have given additional evidence for the relationship between LIG4 mutations and immunological abnormalities [16,17] and revealed new mutations in patients with Dubowitz syndrome or myeloma [18,19]. 

Lig4 disruption led to a lethal phenotype in embryonic mice [20,21]. In contrast, the *Drosophila* and fungal mutants were viable. The *Drosophila* mutants had hypersensitivity to radiation [3], while the budding yeast mutants showed impaired hyphal growth and cell division [2,22,23], unlike the *Magnaporthe grisea*, *Monascus ruber*, or *Mortierella alpina* mutant cells, which showed no defect in sexual or asexual growth [5,24,25].

These various phenotypic changes and sometimes contradictory data are also interesting because this gene is frequently deleted in fungi to increase the frequency of homologous recombination (HR) and fidelity of gene targeting, which is a commonly used technique for studying gene functions [10,24,25,26,27,28,29]. 

Since we were interested in hyphal growth, we wanted to use the dimorphic and attractive model organism, *S. japonicus*, for genetic research [30]. However, during the first mutant isolations, we noticed that the *S. japonicus* cells could produce non-homologous recombinant colonies after integrative transformation [31]. Thus, we aimed to create a *lig4*-disrupted strain that could be suitable for effective gene targeting. However, first of all, we wanted to clarify whether there are physiological side effects caused by the *lig4* disruption or not.

Our results revealed that although disruption of the *lig4* gene improved the gene targeting, the mutant cells had a pleiotropic phenotype, and several cell processes were disturbed.

## 2. Materials and Methods

Yeast strains used in this study: wild-type *S. japonicus var. japonicus* strain, the h^90^ (CCY-44-5-1, CBS354, ATCC10660) (number: 7–1) [32] control strain, and the *lig4*::KanMX6, h^90^ mutant strains (333, 334, 335) (which were isolated from the wild-type *S. japonicus* strain) were used for the experiments.

Media: YEA (2% glucose, 1% yeast extract, 2% agar), YEL (YEA without agar), and YPA (2% glucose, 1% yeast extract (Scharlau), 1% casein tryptone (Scharlau), 2% agar) were used as standard culture media. After transformation, the cells were spread on YEA + 400 µg/mL G418 or YEA + 100 µg/mL Nourseothricin. Minimal media SMA [33] and EMMA were used for the investigation of hyphal growth [34]. YEA + 5 mM caffeine was used for the investigation of stress sensitivity.

Construction of the *lig4*::KanMX6 disruption cassette: The *S. japonicus lig4* (SJAG_02527) gene was amplified from genomic DNA of the wild-type strain with NEB Phusion High Fidelity polymerase using the 529–532 PCR primers (Table S1) and the following parameters: 98 °C 3 min, 98 °C 30 s, 55 °C 30 s, 72 °C 3 min (30 cycles), 72 °C 10 min, and 4 °C ∞.

The KanMX6 cassette was amplified from the pSJK11 plasmid with NEB Phusion High Fidelity polymerase using the 523–524 primers (Table S1) and a 98 °C 3 min, 98 °C 30 s, 60 °C 30 s, 72 °C 1 min 30 s, (30 cycles), 72 °C 10 min, and 4 °C ∞ PCR protocol [35]. 

The *lig4* PCR fragment was cloned into the cloning site of the pJET1.2 vector (Thermo Fisher Scientific, Waltham, MA, USA) according to the manufacturer’s protocol. This DNA construction was transformed into DH5α competent *E. coli* bacterial cells with the heat shock method [36]. The plasmid was isolated from the bacterial cells, and the *lig4* gene was digested with the StuI restriction enzyme (Thermo Scientific, Waltham, MA, USA). The amplified KanMX6 cassette was inserted into the StuI site of the *lig4* gene, containing the pJET1.2 vector.

Integrative transformation of the wild-type *S. japonicus* cells: The cells were transformed by the electroporator method (Gene Pulser Xcell-BioRad, Dubai, United Arab Emirates), according to the manufacturer’s *S. pombe* protocol. The cells were cultured in 100 mL YEL at 30 °C for 16 h and centrifuged (3000 rpm, 5 min). Fresh YEL + hydroxyurea (final concentration was 13 mM) was added to the pellet, and the cells were incubated at 30 °C for 2 h without shaking. After centrifugation (3000 rpm, 5 min, 4 °C), the pellet was washed with MQ water and three times with 1 M sorbitol. After centrifugation, the cells were suspended in 150 µL 1 M sorbitol, and 5 µL of about 1200 ng/µL *lig4:*:KanMX6 DNA construction was added to them. 

Colony PCR for checking homologous recombination: The colony PCR method uses lysed cells instead of extracted DNA. We used a 10 µL pipette tip to pick up a small amount of yeast. We used a microwave oven to lyse the cells, and the heat treatment was carried out with a 1600 W microwave oven (60% power, for 3 min). Then we added the PCR mix immediately to the heat-treated cells.

Genome sequencing to determine the copy number of the KanMX6 cassette: The genomic DNA was isolated from one G418-resistant colony (which proved to be homologous recombinant by colony PCR) (334) with the glass bead method [36]. Library preparation was performed using the tagmentation-based Nextera DNAFlex Library Prep Kit (Illumina, San Diego, CA, USA) according to the manufacturer’s protocol. Paired-end 150 bp sequencing was executed on an Illumina NextSeq 500 instrument. Raw sequencing reads were aligned to the *S. japonicus* sequence [37] using the Burrows–Wheeler Aligner (BWA) algorithm. Genetic variants (single nucleotide polymorphisms, mutations, and indel variants) were determined using the GATK algorithm (https://software.broadinstitute.org/gatk/, accessed on 20 June 2022). A de novo assembly was created with SPAdes v3.14.3 to avoid discarding the integrated KanMX6 cassettes. Thereafter, a BLASTn search was performed on the created contigs to verify the integration and check the copy number of the KanMX6 cassette. Library preparations, sequencing, and data analysis were performed at the Genomic Medicine and Bioinformatics Core Facility of the University of Debrecen, Hungary.

The efficiency of gene targeting in the *lig4* mutant strain: To test the efficiency of gene targeting, the wild-type and the *lig4*::KanMX6 cells (334) were transformed with a linear DNA fragment prepared by an overlapping PCR reaction. This DNA fragment contained a Nourseothricin (NatMX) cassette as a selective marker (https://www.addgene.org/74215, accessed on 16 May 2022), and the 565 and 583 bp long flanking sequences of the SJAG_03918 *S. japonicus* gene, which encodes a hypothetical DNA-binding transcription factor (https://www.japonicusdb.org, accessed on 13 October 2021). For PCR amplification of the flanking sequences, the 1306–1307 and 1305–1309 primers (Table S1) and the following parameters were used: 95 °C 3 min (95 °C 30 s, 58 °C 30 s, and 72 °C 30 s) (30 cycles). The NatMX cassette was amplified from the pMZ379 (Addgene) plasmid with the 1304–1308 primers and the following parameters: 95 °C 3 min, 95 °C 30 s, 60 °C 30 s, and 72 °C 30 s (30 cycles). The overlapping PCR was carried out with the 1306–1309 primers. After transformation, randomly selected Nursethricin-resistant colonies were tested by colony PCR. The percentage of homologous recombinants was calculated by dividing the number of homologous recombinants by the number of total tests. The results are the mean values of two separate experiments. 

Cell morphology test: The mutant (334) and wild-type control strains were streaked onto the surface of the YEA medium, which was incubated at 30 °C, and the morphology of cells was investigated under an Olympus BX-40 microscope after 1 and 2 days. The same experiment was also carried out with YEA + 5 mM caffeine medium. The cell morphology of 700 randomly selected cells per strain was checked. The percentage of chains was calculated by dividing the number of chains by the total cell number. The results are the mean values of two separate experiments.

Hyphal growth: Cell suspension was prepared from a 1 day old culture of the *S. japonicus* cells (333, 334, 335) with MQ water (4 × 10^6^/cell/mL). A quantity of 20 µL of cell suspension was dropped onto the surfaces of YEA, EMMA, and SMA media. The Petri dishes were incubated at 25 and 30 °C. The hyphal growth was photographed after 10 days. The length of hyphae produced on YEA at 30 °C was also measured by a ruler (in 6 positions/drops). The results are the mean values of three separate experiments. 

Sporulation capacity: The cells were streaked on YEA complete medium, and the Petri dishes were incubated at 30 °C. The spores, asci, and cells were counted after 5 days. The percentage of sporulation was calculated by dividing the number of asci and free spores by the total number. The results are the mean values of three separate experiments.

Heat shock treatment: Cell suspension was prepared with MQ water from a 1 day old YEA culture. The initial optical density was OD_595_ = 0.1. For the heat shock experiments, 10× dilutions were prepared. The cells were exposed to 42 °C for 5 and 10 min. After the heat shock, the cells were plated on YEA medium, and the agar plates were incubated at 30 °C. The colonies were counted after 4 days.

UV treatment: To investigate the effect of UV on cells, cell suspensions were prepared with MQ water. The initial concentration of the wild-type and *lig4* mutant strains was set to OD_595_ = 0.1, and then a 10x dilution was prepared. A quantity of 100 μL of the cells was spread on the YEA culture medium, and then they were exposed to UV light (245 nm) for 20, 40, and 60 s. The plates were incubated at 30 °C for 2 days, and the number of colonies was counted. 

Spot assay for testing of caffeine sensitivity: The wild-type and mutant (334) cells were grown at 30 °C in YPL overnight. A quantity of 20 µL of the undiluted culture (OD_595_ = 0.2) and its dilutions (10×, 100×, and 1000×) were spotted on YPA supplemented with 5 mM caffeine. The plates were incubated at 25 °C, and the growth of cells was investigated after 3 days.

Investigation of chronological aging: The wild-type and mutant strains (334) were inoculated into YEL complete liquid medium (OD_595_ = 0.2). The cell suspensions were statically incubated at 30 °C for 5 weeks. At the end of incubation, the cells were spread on the surface of YEA, and the colonies were counted after 5 days. To confirm the results, another mutant strain (333) was also tested. The cells of the 4 week old culture (YEA) were stained with methylene blue solution, and the morphology and color of the cells were checked under an Olympus BX40-DP42 microscope. The living and blue/dead cells were also counted.

Investigation of the mitotic cell cycle: To determine the time necessary for septum degradation, a time-lapse analysis was carried out. Log-phase cells (334, 7–1) were spread onto the surface of YEA, covered by a microscope coverslip, and incubated at 30 °C. Photos were taken every 2 min (Olympus BX40-DP42 microscope, DP Controller software). We determined the time between the appearance and degradation of the septum in 34 mutant and 34 wild-type cells. 

The size of the cells cultured on YEA at 30 °C was measured under the BX40-DP42 microscope. 

RNA sequencing: To obtain global transcriptome data, high-throughput mRNA sequencing analysis was performed on the Illumina sequencing platform. The quality of the total RNA samples was checked on an Agilent BioAnalyzer (Santa Clara, CA, USA) (Eukaryotic Total RNA Nano Kit, according to the manufacturer’s protocol). Samples with an RNA integrity number (RIN) value of 7 were accepted for the library preparation process. The NEBNext Ultra II RNA Sample Prep Kit (New England BioLabs, Ipswich, MA, USA) was used to prepare the library, and sequencing was performed on an Illumina NextSeq 500 machine using the NextSeq 500/550 High output sequencing reagent (75 cycles) with a single read 75 bp readout. The raw data were aligned to the reference genome from EnsemblFungi (https://fungi.ensembl.org/info/data/ftp/index.html, accessed on 22 July 2022) using the Hisat2 algorithm. The StrandNGS program was used for further analysis, and normalization was performed using the Deseq algorithm of the program. The statistical test was performed by a moderated t-test with Benjamini–Hochberg FDR.

The library preparations and the sequencing run were performed by UD-GenoMed Kft and the Genomic Medicine and Bioinformatics Core Facility of the Department of Biochemistry and Molecular Biology, Faculty of Medicine, University of Debrecen, Hungary. 

Bioinformatics analyses: The experimentally characterized and validated Ligase 4 protein sequence DNL4 of *Saccharomyces cerevisiae* was obtained from the Saccharomyces Genome Database (SGD) (https://www.yeastgenome.org/, accessed on 13 January 2023). This sequence was used for a BLASTp search in the database of non-redundant protein sequences at NCBI to identify the putative ligase orthologues of the fission yeasts. LIG4 orthologous protein sequences of *Candida albicans* and *Homo sapiens* were obtained from the Candida Genome Database (http://www.candidagenome.org/, accessed on 13 January 2023) and from the NCBI (https://www.ncbi.nlm.nih.gov/genome?term=human&cmd=DetailsSearch, accessed on 13 January 2023), respectively.

To precisely identify the DNL4 (LIG4) orthologous protein sequence of *S. japonicus*, a phylogenetic tree was built. The tree was created at the website of Phylogeny.fr (http://www.phylogeny.fr/, (accessed on 13 January 2023) using the obtained protein sequences of the concerned species [38]. The sequences were submitted to a manually adjusted workflow consisting of MUSCLE for alignment, GBLOCKS for the curation of the alignment, and PhyML with the WAG substitution model for the phylogeny [39,40,41]. The number of substitution rate categories was adjusted to 4, and the gamma distribution parameter and proportions of invariable sites were both estimated. Branch support was estimated with aLRT analysis [42]. The tree was displayed with FigTree v1.4.2 (http://tree.bio.ed.ac.uk/software/figtree/, accessed on 13 January 2023).

Information on gene structure (exon-intron positions) and protein structure, as well as gene function and GO categories, originated from JaponicusDB (https://www.japonicusdb.org/gene/SJAG_02527, accessed on 13 January 2023) [37]. 

Statistical analyses: The normal distribution of the data was tested by the Shapiro–Wilk test. In the case of normal distribution, one-tailed t tests were used to assess the significant discrepancy between the samples; otherwise, the Mann–Whitney U test was used. For datasets that proved not to be normally distributed, Kruskal–Wallis tests were used for multiple comparisons, followed by Bonferroni-corrected pairwise Dunn tests as post hoc tests. *p* values were considered significant below the alpha level of 0.05. All statistical analyses were performed with the Past v4.09 program [43].

## 3. Results

### 3.1. Identification of the lig4 Putative Orthologue of S. japonicus

**Figure 1:**
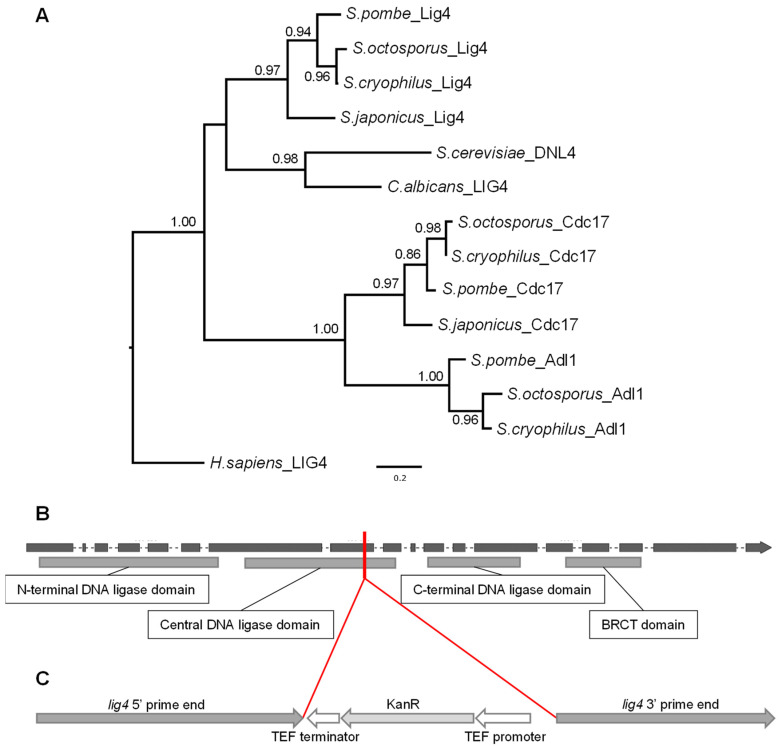
Maximum likelihood phylogenetic tree of DNA ligases to precisely identify the putative orthologue of DNA Ligase 4 (Lig4) in *S. japonicus*. (**A**). The tree indicates that the Ligase 4 sequences unequivocally split from the other DNA ligase sequences. Branch supports came from aLRT analyses; values less than 0.50 are not shown. *H. sapiens* LIG4 was used as an outgroup. The *S. japonicus lig4* gene structure shows that it belongs to the intron-rich genes (**B**). Grey rectangles are the exons; dashes are the introns. The lower rectangles indicate the identified protein domains (according to PFAM). The red line and (**C**) show the integration position of the disruption cassette. The figures are not to scale.

It is known that the NHEJ machinery is conserved throughout the different organisms. One of the key components of NHEJ is the DNA Ligase 4 (LIG4) enzyme. Since the fission yeasts have several different ligase-coding genes according to PomBase and JaponicusDB, we wanted to identify precisely the putative orthologous sequence in *S. japonicus*. In order to do that, we searched for the experimentally validated LIG4 (Dln4p) sequence of *S. cerevisiae* in the SGD. Then we performed BLASTp searches in the NCBI database using the Dln4 protein sequence as a query. The best-scoring hit was the *S. japonicus* sequence SJAG_02527, which is a putative LIG4 orthologue. For further verification, we created a phylogenetic tree using all the ligase sequences of fission yeast that we found with the BLAST searches. We also included the *Candida albicans* and *Homo sapiens* LIG4 protein sequences. The tree indicated that the putative LIG4 orthologous sequences unequivocally split from the other ligase sequences (Figure 1A). Thus, we considered the SJAG_02527 sequence a valid LIG4 orthologue. 

Further sequence analyses have revealed that the *lig4* gene interestingly belongs to the intron-rich genes as it contains 17 introns (only 9 genes from the 4896 have more than 10 introns according to the JaponicusDB) (Figure 1B and Table S2) and is localized to one of the ancestral locally collinear blocks (aLCB) of the fission yeasts [44].

### 3.2. Isolation of the lig4 Mutant Strain

Since our previous results had shown that ectopic integration could occur in *S. japonicus* [31], we decided to prepare a *lig4* mutant strain, which could be suitable for effective gene targeting if the mutation does not cause any side effects. To obtain a *lig4* mutant strain, we prepared the *lig4*::KanMX6 construction (Figure 1C), which was transformed into the wild-type strain. After transformation, randomly selected G418-resistant colonies were isolated (333, 334, and 335). Their colony PCR analyses proved that the *lig4* gene is disrupted in their genomes. One of the colonies (334) was also sent for genome sequencing, which proved that there is only one KanMX6 cassette in its genome.

### 3.3. Phenotypic Analyses of the lig4 Mutant Cells Revealed That They Have Increased Sporulation Capacity on Complete Medium, Decreased Hyphal Growth, and Tend to Produce Chains of Cells in the Presence of Caffeine

**Figure 2:**
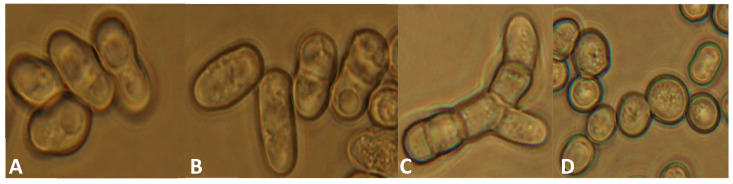
Cell morphology of the *lig4::KanMX6 S. japonicus* cells was normal in complete medium (**A**) (YEL, incubated in a shaker at 25 °C for 4 days) (334); similar morphology was found on solid YEA medium, like the wild-type cells (**B**). However, the mutant strain tends to produce chains of cells on 5 mM caffeine-containing YEA (after 2 days, at 30 °C) (**C**), in contrast to the wild-type cells, which showed normal morphology. The chains disappeared after 7 days (**D**). (The cells were visualized with Nomarski optics).

First of all, we wanted to reveal whether the mutation had caused any side effects or not. The cell morphology and cell division of the mutant strains were investigated. The *lig4*::KanMX6 mutant cells showed normal morphology (Figure 2A), which was similar to the wild-type strain (Figure 2B) on complete medium (YEA and YEL) at 25 and 30 °C. However, the cells seemed to be shorter. When we measured the cells, it confirmed that the size of the mutant cells was a little bit shorter on YEA at 30 °C (Figure S1A,B). In addition, we determined the time necessary for septum degradation through time-lapse analysis. According to our data, the time of septum degradation did not differ significantly on YEA at 30 °C compared to the control (Figure S1C). However, when the cells were grown on a caffeine-containing medium (YEA + 5 mM caffeine) (caffeine can influence the mitotic checkpoints and increase the cytokinesis defects if there are any) [45], 5% of the mutant cells showed pseudo-mycelial morphology (chains of 4–6 cells) after 1–2 days (Figure 2C and Figure S2), while the control culture did not contain any chains. Interestingly, the cells of the chains have separated after 5–7 days of incubation (Figure 2D). 

**Figure 3:**
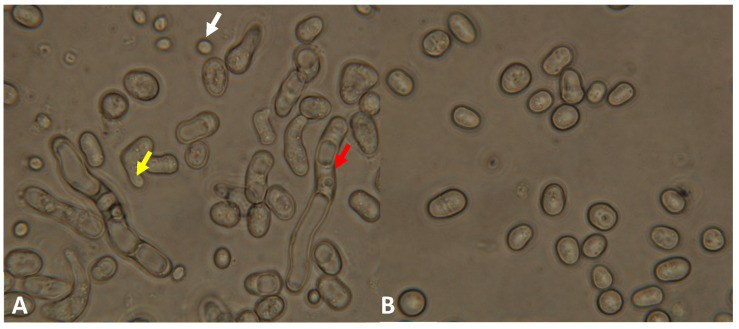
The mutant strain (334) produced cells with conjugation tubes (yellow arrow), spores (white arrow), and longer cells/hyphae (red arrow) on complete YEA medium after 5 days (incubated at 30 °C) (**A**), in contrast to the wild-type strain (**B**), which had only vegetative cells. (The cells were visualized with Nomarski optics).

Investigation of meiotic ability revealed that the mutant strain can form asci. However, we noticed later that the mutant cells tended to conjugate and sporulate even on a complete medium. A 5 day old culture of the mutant strain contained a lot of cells with conjugation tubes, zygotes, and spores on YEA (Figure 3A), in contrast to the normal vegetative cells of the wild-type strain (Figure 3B). 

To obtain further evidence for the higher sporulation frequency, the zygotes and spores were counted. This confirmed that the *lig4* mutant strain produced spores and zygotes in higher numbers on the YEA medium compared to the control (Table 1).

**Figure 4:**
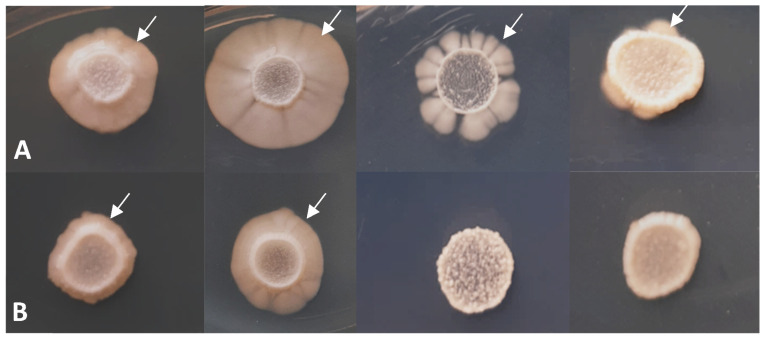
Hyphal growth has decreased in the mutant strain (334) (**B**), compared to the wild-type strain (**A**) (after 10 days on YEA 25 °C, YEA 30 °C, SMA 30 °C, and EMMA 30 °C (from left to right)). White arrows show the hyphae.

Since *S. japonicus* belongs to the dimorphic species and can form hyphae under special growth conditions [46,47,48,49], its hyphal growth was also tested. Figure 4 demonstrates that the *lig4* disruption caused decreased hyphal growth both on complete (YEA) and minimal media (SMA and EMMA) (Figure 4B) compared to the control strain (Figure 4A). The length of hyphae was also measured (YEA, 30 °C), and significant differences were found in all three mutant strains (333, 334, and 335) after the statistical analyses (Figure S3).

### 3.4. The Study of Stress Response Revealed Increased Chronological Aging and Sensitivity to Heat Shock, UV Light, and Caffeine

The altered cell morphology on the caffeine-containing medium (Figure 2C) resembled the mycelial mutants of the closely related *S. pombe*, which were frequently sensitive to stress factors such as ions and drugs [50,51,52]. Therefore, the stress response of the *lig4* mutant cells was also investigated. Our data revealed that the survival of the mutant cells and their colony-forming capacity were lower after heat shock and UV treatment (Table 2 and Figure S4). 

**Figure 5:**
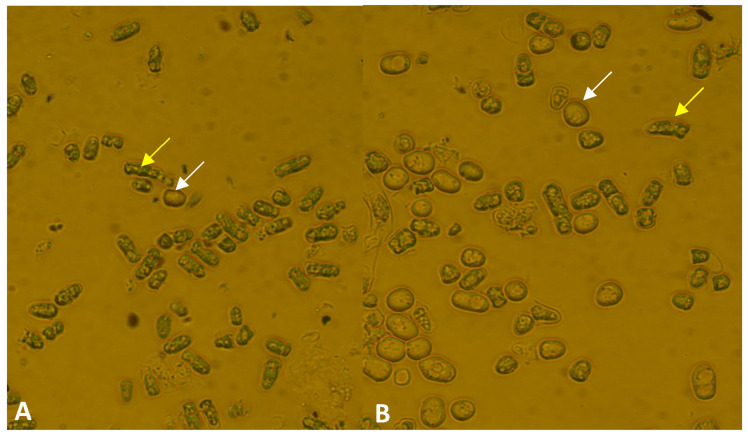
The ratio of dead cells was higher in the 4 week old culture of the *lig4* disrupted strain (**A**) (333) compared to the wild-type strain (**B**). The cells were incubated at 30 °C for 4 weeks on YEA. (The white arrows show the living cells; the yellow arrows show the dead cells).

We also noticed that older cell cultures of the mutant strains (4 or 5 week old cultures) contained a higher number of dead cells. To obtain further information about the aging of the strains, 5 week old cells were spread on a fresh complete medium, and their colony-forming capacity was investigated. Only 12% of the cells produced colonies in the mutant strain (334), compared to 17% in the wild-type strain (one-tailed *t*-test, *p* = 0.0142). Similar results were obtained when we stained the 4 week old cells of the 333 mutant strain with methylene blue. It confirmed our previous data because a higher ratio of dead cells was found in the mutant strain (333) (91%), compared to the wild-type strain (50%) (Figure 5). 

**Figure 6:**
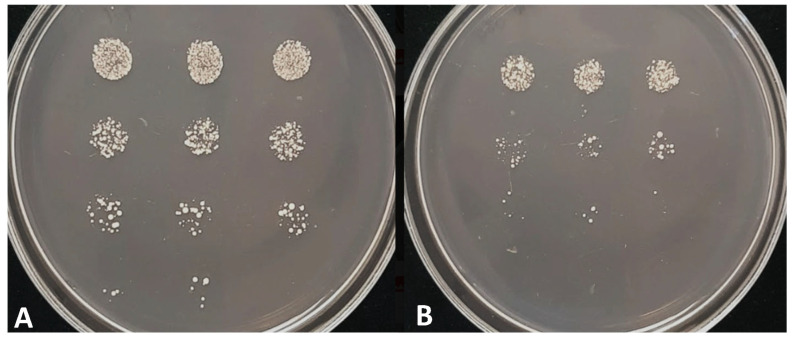
Growth on 5 mM caffeine-containing YEA. The wild-type cells (**A**) and *lig4* disrupted cells (334) (**B**). The agar plates were incubated at 25 °C for 3 days. Cells of the OD_595_ = 0.2 culture and its 10×, 100×, and 1000× dilutions (from top to bottom) were dropped on the agar plates.

In addition, the spot assay showed caffeine sensitivity in the mutant cells (Figure 6).

### 3.5. Higher Flocculation Capacity

**Figure 7:**
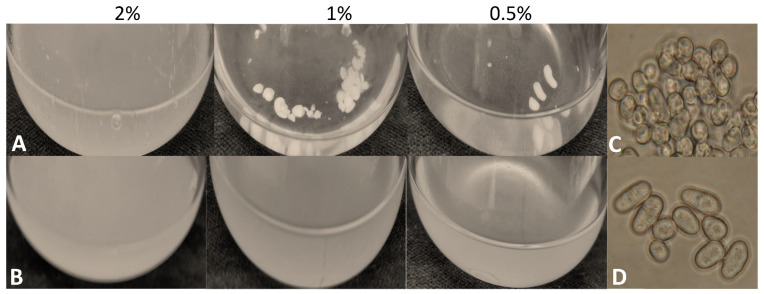
The mutant cells (334) flocculated in liquid medium (YEL) prepared with 2%, 1%, or 0.5% glucose concentrations (**A**) after 5 days. The Erlenmeyer flasks were incubated at 30 °C in a shaker. The flocs contained agglutinated cells (**C**), in contrast to the wild-type cells (7–1) (**B**,**D**).

During breeding of the *S. japonicus* cells, we noticed that the mutant cells tended to flocculate at lower glucose concentrations (1 and 0.5%) (Figure 7A). Microscopic analysis suggested that the cells were highly agglutinated in the flocks (Figure 7C), in contrast to the control strain (Figure 7B,D).

### 3.6. Transcriptional Profiling Confirmed the Phenotypic Changes of the Mutant Strain

To confirm the pleiotropic phenotype of the mutant strain (334), its transcriptional profiling was carried out. The RNA sequencing revealed that 143 genes were downregulated and 257 were upregulated compared to the wild-type strain (their log_2_ fold change values were +/− 1.5 or higher). The heatmap constructed from the normalized values can be found in Figure S5A. Mainly the genes of metabolic processes, transport, and ribosome biogenesis were changed (Table S3).

Confirming the higher sporulation capacity of the mutant strain (Figure 3 and Table 1), most of the genes belonging to ascospore formation (GO:0030437) and meiotic nuclear division (GO:0140013) were upregulated (Table S3). The stronger flocculation (Figure 7C) may originate from the upregulation of the cell adhesion (GO:0007155) genes. In addition, 16 DNA recombination (GO:0006310) genes and 11 repair genes (GO:0006281), among them the Xrc4 (SJAG_02237) (XRCC4 homologous gene) involved in non-homologous end joining, were also upregulated. We have also noticed that the mRNA level of the 3′ part of the *lig4* gene was also increased (Table S3 and Figure S5B), while the neighboring genes of *lig4* did not show changes. 

Unexpectedly, mRNA levels of several genes involved in signaling (GO:0023052) were also changed, such as *mei2* and *mei4*, whose *S. pombe* homologous genes were regulated by *tor2* (Table 3). When we compared the *S. pombe tor2*-induced genes [53] with our results, we found further common genes (Table 3).

### 3.7. Gene Targeting Was Higher in the lig4 Mutant Strain

Since lack of the *lig4* gene often improves the efficiency of gene targeting in fungi [4,25,26,40], we tested this feature of our mutant strain. A linear DNA fragment was prepared, which contained the 565 and 583 bp long UTR regions of a gene encoding an *S. japonicus* hypothetical transcriptional factor (SJAG_3918) and the Nourseothricin resistance gene as a selective marker. This linear DNA fragment was transformed into the *lig4* mutant (334) and the control strains. These cells were then spread on YEA medium supplemented with Nourseothricin, and the number of transformant colonies was counted. We obtained more Nourseothricin-resistant colonies in the mutant strain (334) (average 167 colonies/agar plate from two experiments) than in the case of the wild-type strain (average four colonies/agar plate), although the initial cell number was similar in the two strains. The localization of the NatMX marker gene was checked by colony PCR with sequence-specific primers. It showed that the ratio of homologous recombination has also increased by about 30% in the *lig4* mutant.

## 4. Discussion

In this study, we have successfully disrupted the *S. japonicus lig4* gene (DNA repair ligase, SJAG_02527) and isolated *lig4* mutant strains. They were checked by colony PCR, and the genome of one strain (334) was also sequenced. These experiments have shown that there is only one KanMX6 cassette (used for disruption) in the genome, and its localization is correct. 

The study of the mutant cells revealed that the *lig4* disrupted cells were viable, in contrast to the mice cells [21] and similarly to several fungal mutants [2,4,23,24,26,27,57]. The efficiency of its gene targeting was improved similarly to the *lig4* mutants isolated in *Mortierella alpina*, *Candida glabrata*, or other fungal species [4,24,25,26,27]. Based on this result, the *lig4*-disrupted *S. japonicus* mutant strain could be an attractive strain for gene targeting. However, further study of our mutant strains revealed that there are physiological side effects to this mutation. Although mitosis and cell separation of the *lig4* disrupted cells seemed to be normal under commonly used culture conditions, similar to the *Saccharomyces cerevisiae*, *Candida glabrata*, *Schizosaccharomyces pombe*, or *Magnaporthe grisea* mutant strains [2,5,26,57], we noticed that their cells were shorter on complete medium. Our further tests showed increased sensitivity to heat shock and UV treatment, unlike the *Candida* mutant cells, which were not affected by UV light [2,26]. Since caffeine inhibited the growth of the mutant cells and caused the formation of chains, like in the *S. pombe* “sep” mutants [50,51,52], we assume that the mutant cells are more sensitive to caffeine than the control strain. 

Moreover, hyphal growth and meiosis of the mutant strain were also altered. All three mutant strains produced significantly shorter hyphae compared to the control strain, which was in good agreement with the results obtained in *C. albicans*, which indicated that LIG4 protein was required for hyphal growth [23]. As for meiosis, although our mutant cells were able to sporulate, like the *Magnaporthe grisea* or *S. pombe* mutant strains [5,57], the sporulation capacity of the *S. japonicus* mutant cells increased on the complete medium. That is, the cells behaved as if they were starving for N (sporulation of the fission yeasts is induced by N starvation) [58]. Interestingly, sporulation of the *S. cerevisiae* LIG4 mutant was also abnormal; however, it sporulated less efficiently than the control strain [2]. 

In addition, we also noticed that chronological aging had altered. The viability and colony-forming capacity of the 4–5 week old mutant cells strongly decreased. Since the latter process depends mainly on glucose signaling [59,60], we assume that an alteration of glucose sensing could occur. This idea might be supported by the increased flocculation capacity of the *lig4*-disrupted cells. They strongly flocculated, especially at lower sugar concentrations, in contrast to the control strain. These data might suggest unbalanced or damaged nutrient sensing or supply, which can confuse various cell processes. This idea is in good agreement with the fact that mycelial growth, which is strongly regulated by environmental factors, was also altered [31,46,47,49,61,62].

These phenotypic changes were confirmed by the transcriptional profiling data, as the mRNA levels of several genes involved in sporulation, flocculation, metabolic, and transport processes were altered. In addition, we found genes with higher mRNA levels, such as *mei2*, *mei4*, *fus1*, and *ste11*, which are regulated in the closely related species, *S. pombe*, by *tor2*, which is a main component of the TOR pathway (target of rapamycin) [53,63,64,65]. This is in good agreement with the fact that the TOR pathway is the master regulator of growth and starvation responses, sporulation, life span, and aging [53,64,65,66,67,68,69,70,71]. However, the relationship between *lig4* disruption, the TOR pathway, and nutrient supply requires further studies. 

The phenotypic changes can be caused by a truncated protein that disturbs the cell processes (our mutant was a disrupted mutant, which showed a higher mRNA level in the *lig4*’s 3′ region). This can be in connection with the presence of the KanMX6 TEF promoter, similarly to the results of Powers [72]. If that is the case, the KanMX6 cassettes should be used only with a special promoter [72]. Another explanation of the abnormalities can be that a higher mutational rate and a weaker repair mechanism induced by the missing lig4 protein [2] can disturb cellular processes. This possibility can be supported by the fact that there is crosstalk between the processes, e.g., DNA damage response and the TOR pathway [70]. Further evidence can be found for this idea because alteration of mycelial morphogenesis, conidiation, and the stress response has also been obtained in the case of the KU80 mutation [73], and the KU heterodimer functions in various cellular processes [74]. Based on these data, we think that manipulations of the NHEJ-related genes must be avoided because they can have more serious consequences than we expect.

## Figures and Tables

**Table 1:** The sporulation frequency of the *lig4* mutant cells (334) was higher on the complete YEA medium compared to the wild-type strain. The agar plates were incubated at 30 °C for 5 days.

Strain	Number of Spores and Zygotes (%)	
	Spore	Zygote
Wild-type	4.3	0.6
Mutant	10.6	4.7

**Table 2:** The colony-forming capacity of the *lig4* disrupted cells (334) decreased after heat shock and UV light treatment compared to the wild-type cells.

Strain	Colony Forming Capacity (%)		One Tailed *t*-Test *p*-Values
	Wild-Type	Mutant	
Treatment			
0 min heat shock	100	100	1.00
5 min heat shock	99.5	78.5	0.00521
10 min heat shock	93.0	45.6	1.2796 × 10^−6^
0 min UV treatment	100	100	1.00
20 s UV treatment	63.8	42.7	0.00223
40 s UV treatment	21.0	1.6	1.8818 × 10^−6^
60 s UV treatment	2.6	0.1	2.1902 × 10^−5^

**Table 3:** The homologous genes having altered mRNA levels in the *S. pombe tor2* and *S. japonicus lig4* mutant strains.

Gene Identifier in *S. japonicus*	Function in *S. japonicus*	mRNA Level in the *lig4* Mutant Strain	Gene Identifier in *S. pombe*	Gene Name or Function in *S. pombe*	mRNA Level in *S. pombe tor2-s6* [49] Mutant
SJAG_05288	DNA-binding forkhead transcription factor, meiotic Mei4	+	SPBC32H8.11	*mei4*	+
SJAG_00145	RNA-binding protein involved in meiosis Mei2	+	SPAC27D7.03	*mei2* [54,55]	+
SJAG_03405	zf-FYVE type zinc finger protein and glutamine sensor Pib2	−	SPBC9B6.03	zinc finger protein, Pib2	+
SJAG_04987	HbrB family protein involved in TOR signaling Bit2	−	SPAC6B12.03c	*bit2*	+
SJAG_00100	meiosis specific cyclin Crs1	+	SPBC2G2.09	*crs1*	+
SJAG_01978	pheromone p-factor receptor Mam2	+	SPAC11H11.04	*mam2*	+
SJAG_00781	P-factor pheromone Map2	+	SPCC1795.06	*map2*	+
SJAG_04530	Rad22 homolog Rti1	+	SPBC119.14	*rti1*	+
SJAG_02652	linear element-associated protein Hop1	+	SPBC1718.02	*hop1*	+
SJAG_01943	formin Fus1	+	SPAC20G4.02	*fus1*	+
SJAG_02286	dynein heavy chain, minus-end directed microtubule motor Dhc1	+	SPAC1093.06	*dhc1*	+
SJAG_04009	DNA-binding transcription factor Ste11	+	SPBC32C12.02	*ste11* [54,55]	+
SJAG_02237	XRCC4 nonhomologous end joining factor Xrc4	+	SPAC6G9.16c	*xrc4*	+
SJAG_04950	meu13, Tat binding protein 1(TBP-1)-interacting protein (TBPIP) homolog	+	SPAC222.15	*meu13*	+
SJAG_02003	yippee-like protein	−	SPAPJ691.02	yippee-like protein	+
SJAG_05004	mug8, DUF1708 family conserved fungal protein, cell division site	+	SPAC32A11.01	*mug8*	+
SJAG_03644	MFS transporter superfamily	+	SPBC1271.09	*tgp1*	+
SJAG_02016	mitochondrial inner membrane anchored oxidoreductase Aif1	+	SPAC26F1.14c	*aif1*	+
SJAG_03958	xylose and arabinose reductase	+	SPAC2F3.05c	xylose and arabinose reductase	+
SJAG_00138	DUF1774 family multi-spanning conserved fungal membrane protein	−	SPAC637.03	DUF1774 family multi-spanning conserved fungal membrane protein	+
SJAG_00377	*Schizosaccharomyces* specific protein Meu32	+	SPAP27G11.08c	*meu32*	+
SJAG_01869	NADH/NADPH-dependent indole-3-acetaldehyde reductase, implicated in cellular detoxification	−	SPAC19G12.09	NADH/NADPH-dependent indole-3-acetaldehyde reductase, implicated in cellular detoxification	+
SJAG_00167	Toc1, Tor complex Tor2 interacting protein 1	−	SPBP18G5.03	*toc1* [56]	*

+: upregulated; −: downregulated; *: no significant changes.

## Data Availability

Every piece of data generated or analyzed during this study are included in this published article (and its Supplementary Information files).

## Supplementary Materials

The following supporting information can be downloaded at: https://www.mdpi.com/article/10.3390/jof9050550/s1, Figure S1. Cell size and time of septum degradation. Figure S2. Cell morphology on caffeine-containing medium. Figure S3. Length of hyphae. Figure S4. Colony-forming capacity after UV treatment. Figure S5. Heatmap obtained from the RNA sequencing normalized data (A) and coverage of the *lig4* gene (B,C). Table S1: The primers used in this study. Table S2: *S. japonicus* genes with a high intron number. Table S3: RNA sequencing data and GO categories.
