# Supplementary material for: Disruption of the Schizosaccharomyces japonicus lig4 Disturbs Several Cellular Processes and Leads to a Pleiotropic Phenotype
Source: J Fungi (Basel). 2023 May 10;9(5):550. doi: 10.3390/jof9050550 (PMC10219070; doi:10.3390/jof9050550)
Supplement: Supplementary file 1 [file jof-09-00550-s001.zip › Figure S1 cell size, septum degradation.pptx]

## Slide 1
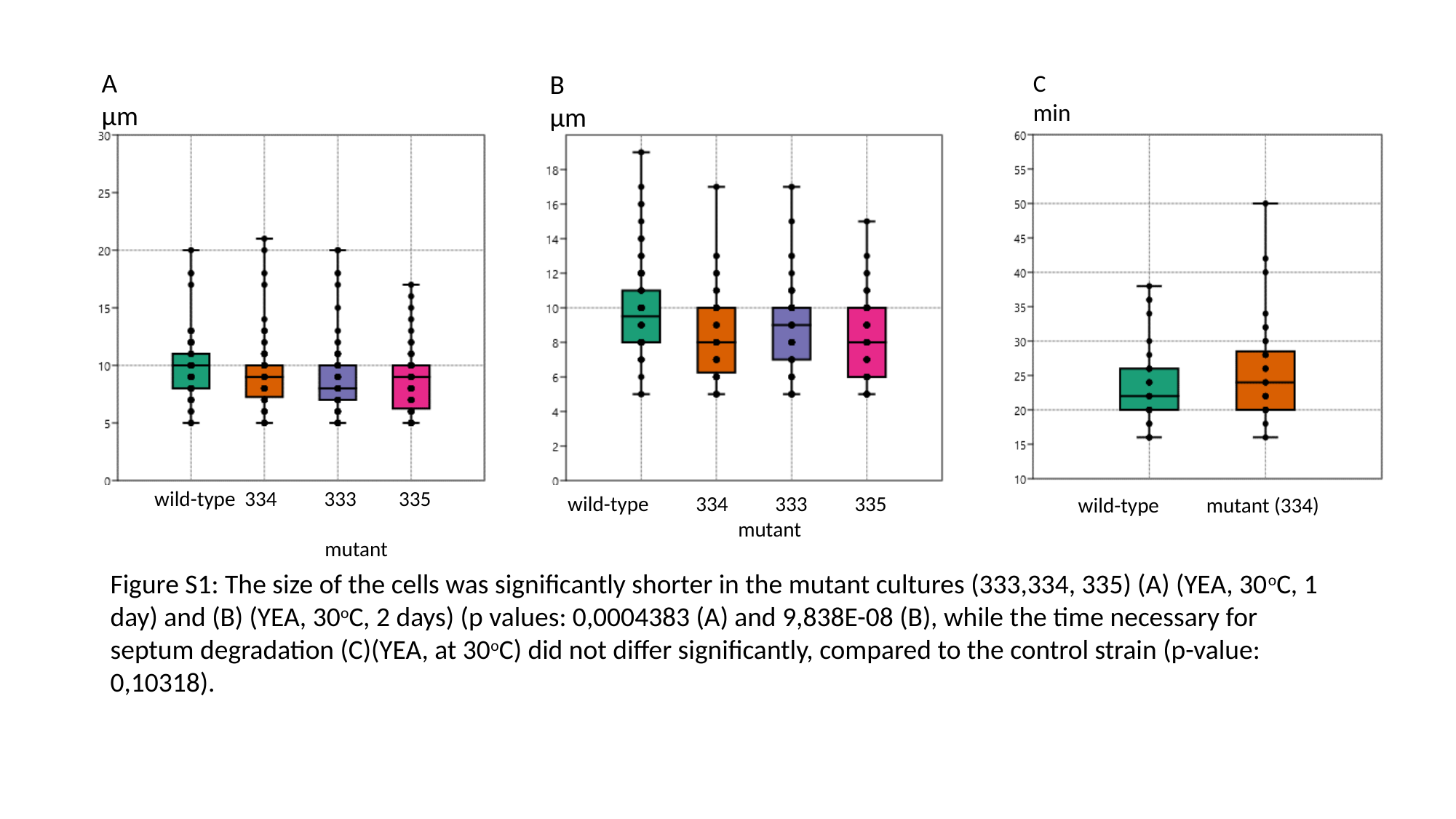

A µm
 wild-type 334 333 335
 mutant
B µm
 wild-type 334 333 335
 mutant
C
min
 wild-type mutant (334)
Figure S1: The size of the cells was significantly shorter in the mutant cultures (333,334, 335) (A) (YEA, 30oC, 1 day) and (B) (YEA, 30oC, 2 days) (p values: 0,0004383 (A) and 9,838E-08 (B), while the time necessary for septum degradation (C)(YEA, at 30oC) did not differ significantly, compared to the control strain (p-value: 0,10318).
