# Supplementary material for: Disruption of the Schizosaccharomyces japonicus lig4 Disturbs Several Cellular Processes and Leads to a Pleiotropic Phenotype
Source: J Fungi (Basel). 2023 May 10;9(5):550. doi: 10.3390/jof9050550 (PMC10219070; doi:10.3390/jof9050550)
Supplement: Supplementary file 1 [file jof-09-00550-s001.zip › Figure S2 Chains on caffeine containing medium.pptx]

## Slide 1
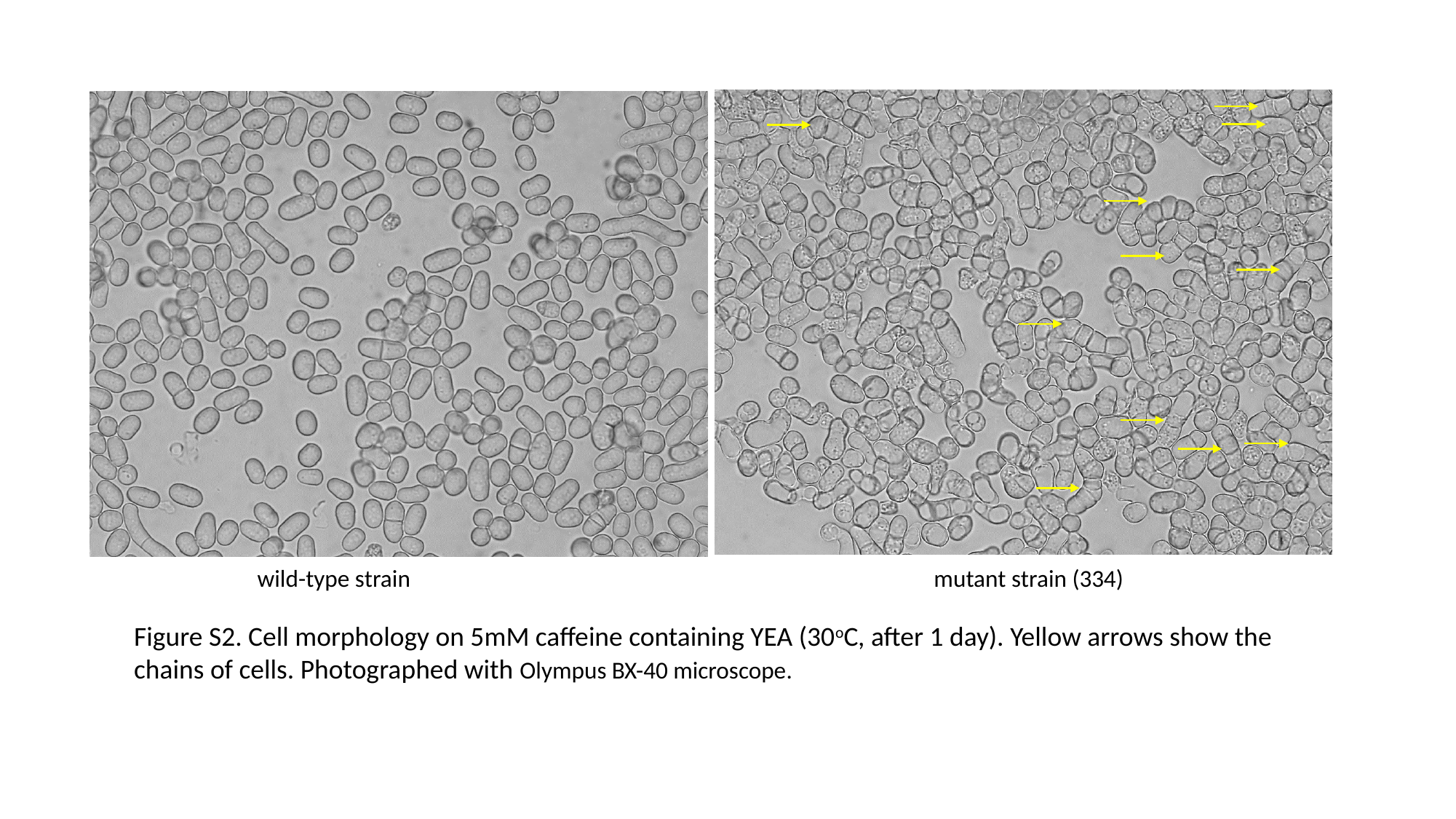

wild-type strain mutant strain (334)
Figure S2. Cell morphology on 5mM caffeine containing YEA (30oC, after 1 day). Yellow arrows show the chains of cells. Photographed with Olympus BX-40 microscope.
