# Supplementary material for: Disruption of the Schizosaccharomyces japonicus lig4 Disturbs Several Cellular Processes and Leads to a Pleiotropic Phenotype
Source: J Fungi (Basel). 2023 May 10;9(5):550. doi: 10.3390/jof9050550 (PMC10219070; doi:10.3390/jof9050550)
Supplement: Supplementary file 1 [file jof-09-00550-s001.zip › Figure S3 Length of hyphae.pptx]

## Slide 1
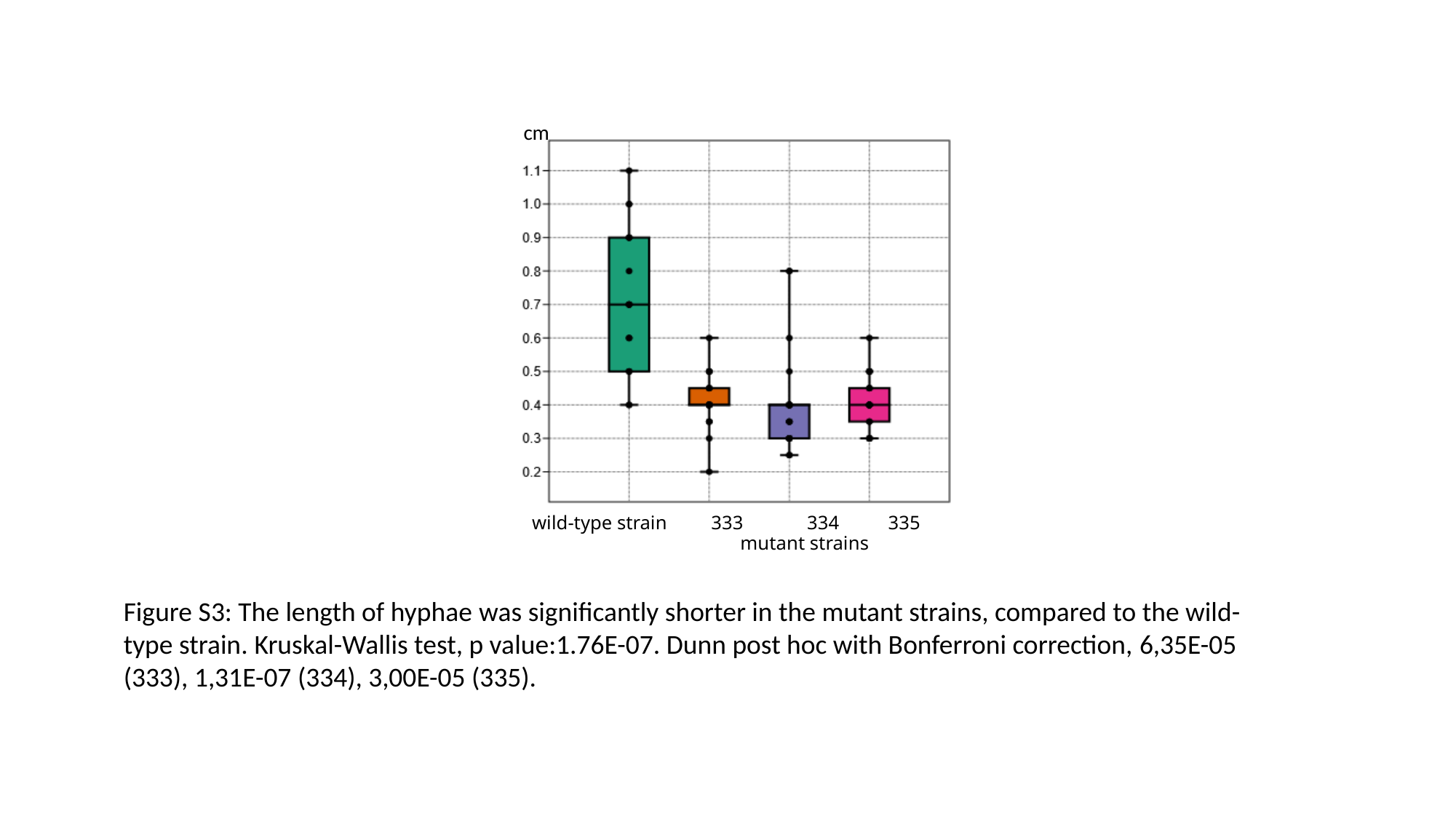

cm
# wild-type strain 333 334 335 mutant strains
Figure S3: The length of hyphae was significantly shorter in the mutant strains, compared to the wild-type strain. Kruskal-Wallis test, p value:1.76E-07. Dunn post hoc with Bonferroni correction, 6,35E-05 (333), 1,31E-07 (334), 3,00E-05 (335).
