# Supplementary material for: Disruption of the Schizosaccharomyces japonicus lig4 Disturbs Several Cellular Processes and Leads to a Pleiotropic Phenotype
Source: J Fungi (Basel). 2023 May 10;9(5):550. doi: 10.3390/jof9050550 (PMC10219070; doi:10.3390/jof9050550)
Supplement: Supplementary file 1 [file jof-09-00550-s001.zip › Figure S4 UV treatment.pptx]

## Slide 1
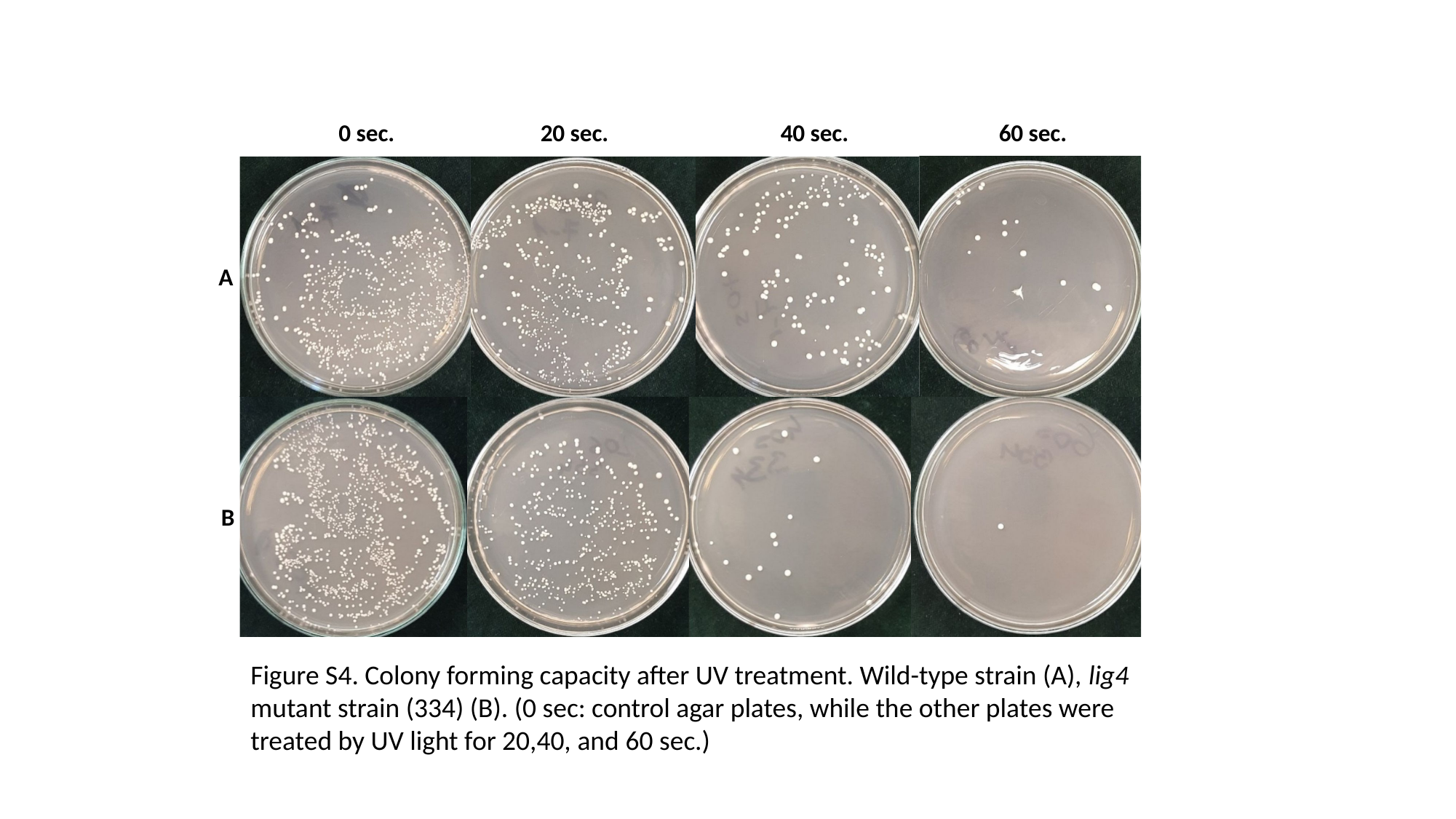

0 sec.	 20 sec.	 40 sec.	 60 sec.
A
B
Figure S4. Colony forming capacity after UV treatment. Wild-type strain (A), lig4 mutant strain (334) (B). (0 sec: control agar plates, while the other plates were treated by UV light for 20,40, and 60 sec.)
