# Supplementary material for: Disruption of the Schizosaccharomyces japonicus lig4 Disturbs Several Cellular Processes and Leads to a Pleiotropic Phenotype
Source: J Fungi (Basel). 2023 May 10;9(5):550. doi: 10.3390/jof9050550 (PMC10219070; doi:10.3390/jof9050550)
Supplement: Supplementary file 1 [file jof-09-00550-s001.zip › Figure S5 heat map, lig4 coverage.pptx]

## Slide 1
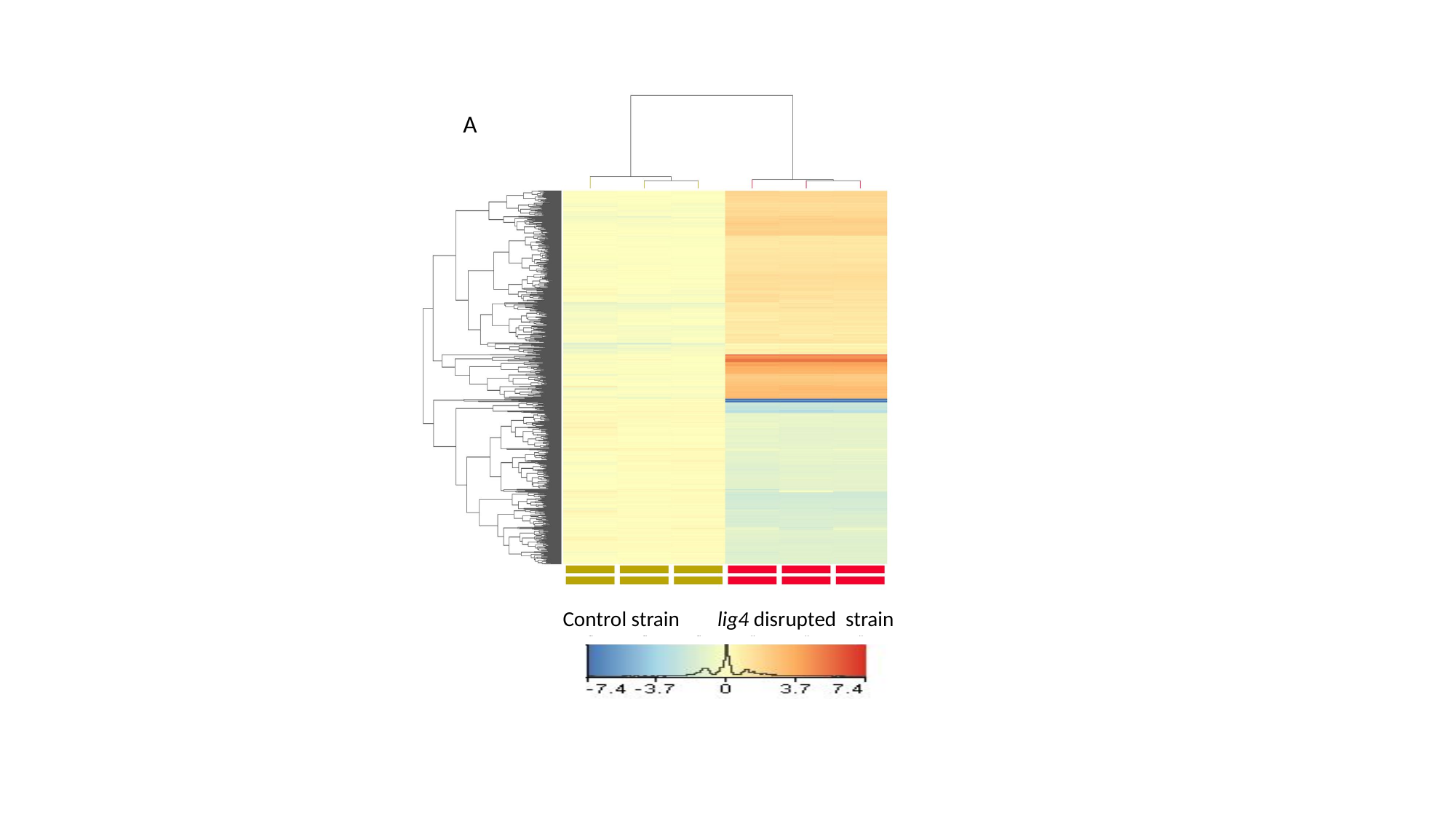

Control strain lig4 disrupted strain
A

## Slide 2
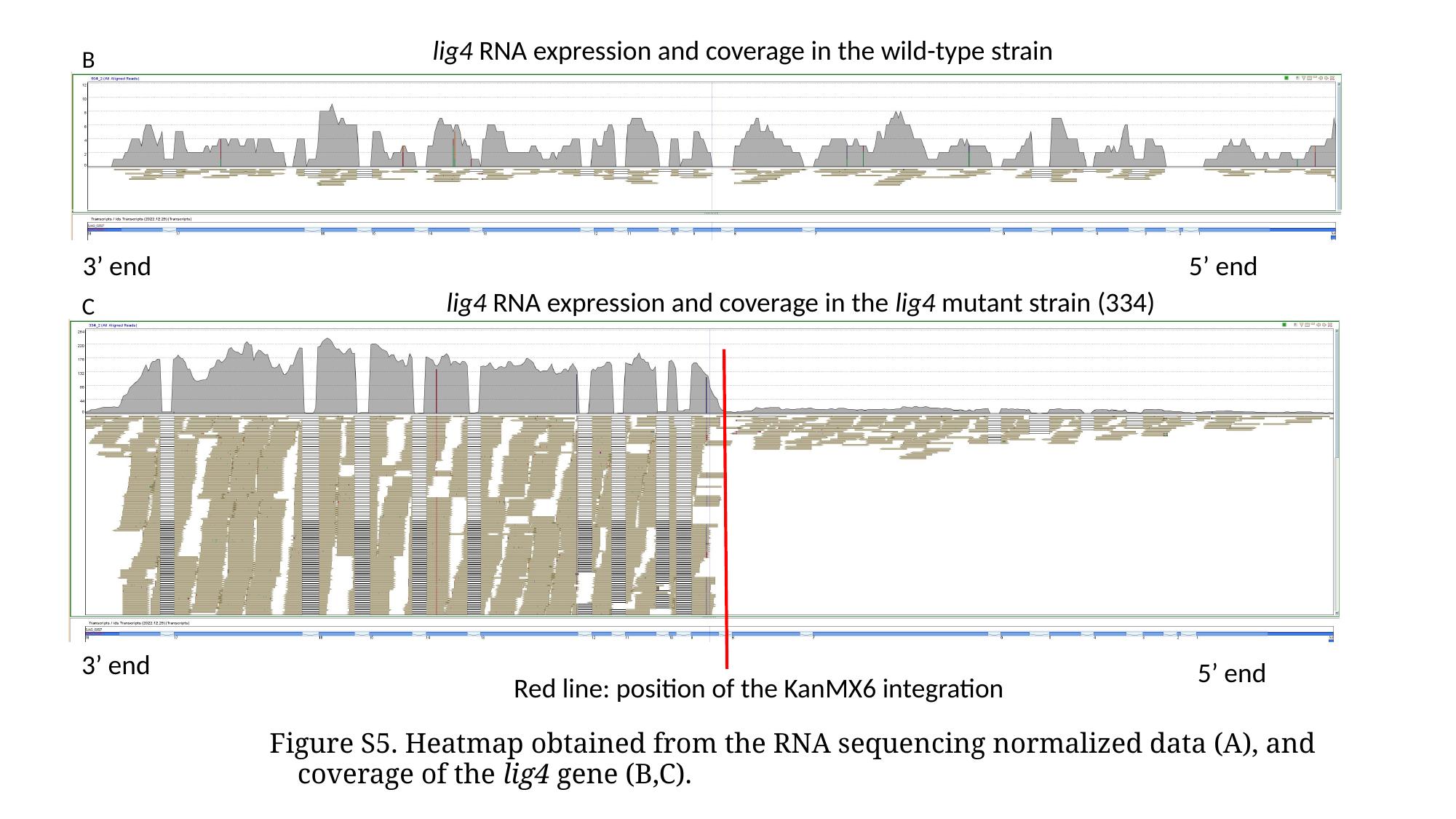

lig4 RNA expression and coverage in the wild-type strain
B
3’ end
5’ end
Figure S5. Heatmap obtained from the RNA sequencing normalized data (A), and coverage of the lig4 gene (B,C).
lig4 RNA expression and coverage in the lig4 mutant strain (334)
C
3’ end
5’ end
Red line: position of the KanMX6 integration
