# Supplementary material for: Disruption of the Schizosaccharomyces japonicus lig4 Disturbs Several Cellular Processes and Leads to a Pleiotropic Phenotype
Source: J Fungi (Basel). 2023 May 10;9(5):550. doi: 10.3390/jof9050550 (PMC10219070; doi:10.3390/jof9050550)
Supplement: Supplementary file 1 [file jof-09-00550-s001.zip › Table S1 primers.pdf]

Number of the primers	Sequence (5′-3′)	Gene
529	CGCAAACGTTAGAATTGCCCAG	*lig4*
532	TGCCCGTCAAGTTTCTGCTTTG	*lig4*
523	CGAGCATGCGTAAAACGACGGCCAGT	KanMX6 cassette
524	GAAGCCGGCGCGTTAGTATCGAATCG	KanMX6 cassette
1306	CGAGTCGGAAGAAGGTCA	SJAG_03918 5′UTR
1307	GTATTCTGGGCCTCCATGTCCACTCTGTTGACAACTCTGC	SJAG_03918 5′UTR
1305	CTGGTCGCTATACTGCTGTCGACATCATTCAGCAATTCG	SJAG_03918 3′UTR
1309	GAGACGGAAGGCAGCAATC	SJAG_03918 3′UTR
1308	GCAGAGTTGTCAACAGAGTGGACATGGAGGCCCAGAATAC	*NatMX* TEF promoter
1304	CGAATTGCTGAATGATGTCGACAGCAGTATAGCGACCAG	*NatMX* TEF terminator
